# Altered Cerebellar Resting-State Functional Connectivity in Early-Stage Parkinson's Disease Patients With Cognitive Impairment

**DOI:** 10.3389/fneur.2021.678013

**Published:** 2021-08-25

**Authors:** Xiaojuan Dan, Yang Hu, Junyan Sun, Linlin Gao, Yongtao Zhou, Jinghong Ma, Julien Doyon, Tao Wu, Piu Chan

**Affiliations:** ^1^Department of Neurology, Xuanwu Hospital of Capital Medical University, Beijing, China; ^2^Key Laboratory on Neurodegenerative Disorders of Ministry of Education, Key Laboratory on Parkinson's Disease of Beijing, Beijing, China; ^3^Laboratory of Psychological Health and Imaging, Shanghai Mental Health Center, Shanghai Jiao Tong University School of Medicine, Shanghai, China; ^4^Department of Neurobiology, Xuanwu Hospital of Capital Medical University, Beijing, China; ^5^Department of Neurology and Neurosurgery, Montreal Neurological Institute, McGill University, Montreal, QC, Canada; ^6^National Clinical Research Center for Geriatric Disorders, Beijing, China; ^7^Advanced Innovation Center for Human Brain Protection, Beijing Institute for Brain Disorders Parkinson's Disease Center, Capital Medical University, Beijing, China

**Keywords:** Parkinson's disease, resting-state fMRI, cognitive impairment, cerebellum, functional connectivity

## Abstract

**Background:** Cognitive impairment is one of the most prominent non-motor symptoms in Parkinson's disease (PD), due in part to known cerebellar dysfunctions. Furthermore, previous studies have reported altered cerebellar functional connectivity (FC) in PD patients. Yet whether these changes are also due to the cognitive deficits in PD remain unclear.

**Methods:** A total of 122 non-dementia participants, including 64 patients with early PD and 58 age- and gender-matched elderly controls were stratified into four groups based on their cognitive status (normal cognition vs. cognitive impairment). Cerebellar volumetry and FC were investigated by analyzing, respectively, structural and resting-state functional MRI data among groups using quality control and quantitative measures. Correlation analysis between MRI metrics and clinical features (motor and cognitive scores) were performed.

**Results:** Compared to healthy control subjects with no cognitive deficits, altered cerebellar FC were observed in early PD participants with both motor and cognitive deficits, but not in PD patients with normal cognition, nor elderly subjects showing signs of a cognitive impairment. Moreover, connectivity between the “motor” cerebellum and SMA was positively correlated with motor scores, while intracerebellar connectivity was positively correlated with cognitive scores in PD patients with cognitive impairment. No cerebellar volumetric difference was observed between groups.

**Conclusions:** These findings show that altered cerebellar FC during resting state in early PD patients may be driven not solely by the motor deficits, but by cognitive deficits as well, hence highlighting the interplay between motor and cognitive functioning, and possibly reflecting compensatory mechanisms, in the early PD.

## Introduction

Parkinson's disease (PD) is the most common neurodegenerative movement disorder characterized clinically by classical motor features including bradykinesia, rigidity, tremor, and postural instability. Yet this disease is also known to lead to a wide range of non-motor symptoms (1, 2). Indeed, cognitive deficits are among the most prominent non-motor features in PD (3), as they are present in as much as 34% of early-stage cases (4, 5) and are detrimental to patients' quality of life (6). Traditionally, the pathophysiological mechanism underlying PD has been linked to the dysfunction of the striato-thalamo-cortical circuitry arising from the degeneration of basal ganglia (BG), the latter accounting partly for the cardinal motor symptoms of PD (7, 8). Driven by the advances of anatomical and human neuroimaging studies in the past 2 decades, however, reciprocal basal ganglia-cerebellar and cerebellar-cortical parcellated networks have been identified, and the role of the cerebellum in PD has received increasing interest (9–16).

Numerous studies have repeatedly suggested that the human cerebellum is not solely processing motor information but also contributes to cognitive functions (14, 17). The existence of a “motor” cerebellum including lobules V, VI, VIIb, and VIII as well as of a “cognitive” cerebellum including crus I and II supports this functional cerebellar dissociation (9, 12, 15, 18, 19). A recent meta-analysis has also reported cerebellar hyperactivations in PD patients who were administered cognitive or motor paradigms with significant cognitive task demands, hence suggesting that one of the main cerebellar implications in PD is linked to cognitive functioning (16). Finally, using structural and resting-state functional magnetic resonance imaging (rs-fMRI), O'Callaghan et al. (20) revealed gray matter loss across the “motor” and “cognitive” cerebellar territories and altered cerebellar functional connectivity (FC) with the cortex in patients with PD compared to normal controls. However, whether such alterations in resting-state cerebellar FC are due to the motor deficits or cognitive deficits in PD patients remains unclear, especially in the early-stage of the disease.

In the current study, cerebellar FC was thus investigated in rs-fMRI data, and both “motor” and “cognitive” cerebellar regions were defined as seeds to shed light on the contributions of motor and cognitive function to the cerebellar connectivity in a group of PD participants in their early phase of disease. We hypothesized that the change of cerebellar FC in early PD was related to both the patients' motor and cognitive deficits. To test this hypothesis, we restricted our recruitment to the early stage of PD patients without dementia, and compared their performance and FC to those of matched elderly control participants. In particular, we stratified the participants into four groups based on their cognitive status: (1) matched elderly control subjects with normal cognition (EC-NC); (2) matched elderly control subjects with evidence of cognitive impairment (EC-CI); (3) PD patients with motor deficits but normal cognitive abilities (PD-NC); and (4) PD patients with both motor and cognitive deficits (PD-CI). Cerebellar gray matter (GM) volumes and seed-to-whole brain connectivity were then, respectively, compared between normal controls and participants with either motor or cognitive deficits alone, or with both types of deficits.

## Methods

### Participants

A total of 122 right-handed subjects, including 64 patients with PD and 58 matched control subjects were asked to participate in this study. Patients with PD were recruited from the Parkinson and Movement Disorder Center at the Xuanwu Hospital of Capital Medical University in Beijing. Clinically established diagnoses of idiopathic PD were made based on the Movement Disorder Society (MDS) clinical diagnostic criteria (1). Scores on the new MDS Unified Parkinson's Disease Rating Scale (MDS-UPDRS) and Hoehn and Yahr (H&Y) scale were attributed to each patient by two separate movement disorder specialists (21). In order to test patients in the early stage of the disease, only clinically established PD cases with H&Y stage ≤ 2 were included in this study. By contrast, the matched control participants were recruited from the “Beijing Longitudinal Study on Aging” community cohort. None of the elderly control subjects had a history of neurological/psychiatric disorders, nor were they taking any psychoactive medications. Participants with dementia were excluded based on the Diagnostic and Statistical Manual of Mental Disorders (*DSM-V*) (22). The current study was conducted in accordance with the Declaration of Helsinki and approved by the Ethic Review Committee of the Xuanwu Hospital of Capital Medical University. Written informed consent was provided by all participants before being enrolled in this study.

### Clinical and Neuropsychological Assessment

Demographic information including date of birth, sex, educational level and cognitive scores, as well as PD clinical characteristics, was obtained. The total daily levodopa equivalent dosage was also calculated for each patient. Specifically, 11 patients were newly diagnosed and unmedicated, while the other 55 patients were either given L-DOPA monotherapy, a dopaminergic agonist, a monoamine oxidase inhibitor, L-DOPA plus entacapone, or a combination of these medications. To minimize the effects of medication on our pattern of results, all clinical measurements and MRI data in all patients were assessed or acquired while they were “off” medication (i.e., no antiparkinsonian medication at least 12 h before examination or scanning).

Mood was assessed using the 17-item Hamilton Rating Scale for Depression (HAMD), and thus, no participants with signs of depression were included in this study (HAMD scores <13) (23). The participant's global cognitive functioning was also measured using the Montreal cognitive assessment (MoCA) Beijing version. Participants without dementia (based on DSM-V criteria) were divided into two subgroups: those who presented mild cognitive impairment (CI group) and those who showed a normal level of cognitive functioning (NC group). The definition of CI was based on established criteria by the National Institute on Aging-Alzheimer's Association (NIA-AA) workshops and the MDS Task Force Level I criteria for mild cognitive impairment (24, 25). Specifically, we employed the following criteria: (i) a complaint of cognitive decline (in the context of established PD for PD-CI); (ii) a deficit in at least one or more cognitive domains and with a MoCA score < 26; (iii) cognitive deficits that are not sufficient to interfere with the patient's independence in day-to-day functions, as assessed with the activities of the daily living (ADL) scale (26); and (iv) absence of dementia (based on DSM-V criteria) (22). As a consequence, the elderly control subjects (EC) were either classified as EC-CI or EC-NC, while patients with PD were classified as part of the PD-CI or PD-NC.

### Neuroimaging Data Acquisition

MRI scans were performed on a 3T MR scanner (Skyra system; Siemens Magnetom scanner, Germany) with a standard 12-channel head coil. The foam padding was used to restrict head motion. High-resolution anatomic images were acquired with a 3D T1-weighted magnetization prepared rapid acquisition gradient echo (MPRAGE) scan [TR = 2,530 ms, TE = 2.98 ms, 192 sagittal slices, slice thickness = 1.0 mm, no gap, field of view (FOV) = 224 × 256 mm, voxel size of 1 mm^3^ isotropic, duration = 313 s]. By contrast, resting-state fMRI data were acquired using a standard gradient-echo echo-planar sequence (TR = 2,000 ms, TE = 30 ms, 35 axial slices, slice thickness = 3 mm, no gap, 176 time points, Flip angle = 90°, FOV = 256 × 256 mm, matrix size = 64 × 64, duration = 360 s). During the resting-state scan, individuals were instructed to keep their eyes closed and relax, but to stay awake. All structural brain MR images were checked for quality control, and subjects exhibiting abnormal brain structures were excluded from the study.

### Structural MRI Preprocessing and Volumetric Analysis

Image segmentation of the cerebellum was performed using the Spatially Unbiased Infratentorial Template toolbox (SUIT Version 3.3) implemented in Statistical Parametric Mapping, version 12 (SPM12, http://www.fil.ion.ucl.ac.uk/spm) and running under MATLAB (R2017a) (The Math-Works Inc., Natick, MA, USA). All images were parallel-aligned to the anterior commissure–posterior commissure line and kept left-posterior-inferior orientation before segmenting the cerebellum. These high-resolution T1 images were then used to isolate the cerebellum from the rest of the brain and normalized to the SUIT template using a non-linear deformation. The outputs were visually inspected for accuracy, and manual edits as well as reprocessing were performed if needed. The gray matter volume of each cerebellar anatomic subdivision from the SUIT probabilistic atlas (27) was extracted for all participants and compared between four different participant groups. The volumes related to the “motor” cerebellum and the “cognitive” cerebellum, which were created using cerebellar subregions from the probabilistic atlas referenced above, were also compared between groups, respectively (15). Furthermore, voxel-based morphometry analysis of the cerebellar gray matter was conducted after being normalized and smoothed using a Gaussian filter kernel with 8 mm full width at half maximum (FWHM) in SPM12. Both ROI- and voxel-based statistical analyses were implemented with significance level at a 2-tailed *p*-value < 0.05, corrected via false discovery rate (FDR) correction for multiple comparisons.

### Functional MRI Preprocessing and FC Analysis

A subsample of participants from the study (43 elderly controls and 50 PD patients) completed an rs-fMRI scan and underwent resting-state functional connectivity analysis. Preprocessing and analyses of resting-state data were conducted using REST plus software (REST plus v1.24_20200725, http://www.restfmri.net) running under MATLAB (R2014b) (The Math-Works Inc.) (28). The preprocessing steps were carried out using the standard pipeline (see Supplementary Methods). Visual inspection was carried out at each step. Seven subjects (three controls and four patients) had excessive head motion (>2 mm and/or 2°) and were thus excluded from further data analyses.

To compare the whole brain functional connectivity patterns of the “motor” and “cognitive” cerebellum between the four patient and control groups, seed-based FC analyses were performed. Region of interest (ROI) masks were created based on the SUIT probabilistic atlas (“motor” cerebellar, CBMm: bilateral lobules V, VI, VIIb, VIIIa and VIIIb; “cognitive” cerebellum, CBMc: bilateral Crus I and Crus II) and resampled, respectively (27). The time series from each cerebellar seed ROI were extracted by averaging signals of all included voxels, and the latter were correlated with the other brain voxels. Pearson's correlation coefficients between seed region and the entire brain were converted using the Fisher's r-to-Z transformation and then statistically compared among the four different participant groups. Multiple comparisons were corrected using REST plus AlphaSim program (REST plus v1.24, http://www.restfmri.net), and Monte Carlo simulations were performed to control Type I error (parameters: individual voxel *p* = 0.01, 10,000 simulations, an estimate FWHM based on statistical map, with 61 × 73 × 61 brain mask, see Supplementary Table 1 for the adopted minimal cluster sizes). We used a corrected significance cluster level of *p* < 0.01. FC measures in significant clusters were extracted for additional statistical analyses.

### Statistical Analysis

To compare the demographic and clinical characteristics of the study participants, we performed chi-square test for categorical variables and two-sample Student *t*-test and/or analysis of variance (ANOVA) for continuous variables. The threshold used for statistical significance was set with Bonferroni corrected *p*-value < 0.05 (SPSS for Windows, Version 21.0; SPSS, Chicago, IL, USA).

Statistical analyses of the imaging data were carried out using the SPM12 and REST Plus software. One-way ANOVA was used to compare differences among the four study groups (EC-NC, EC-CI, PD-NC, PD-CI). Furthermore, stratification analyses were conducted using two-sample *t*-test based on the cognitive (EC-CI vs. EC-NC), motor (PD-NC vs. EC-NC) or combined status with both cognitive and motor functions (PD-CI vs. PD-NC/EC-CI/EC-NC, respectively). To control for the potentially confounding effect of disease severity between PD patients with different levels of cognitive functioning, the disease duration and UPDRS motor scores were adjusted as covariables while comparing the cerebellar FC between PD-NC and PD-CI groups. Multiple comparisons were corrected for volumetric analyses (FDR correction) and cerebellar FC analyses (AlphaSim correction).

ROIs/clusters that were significantly different based upon morphometric and FC analysis were extracted for correlation analyses with clinical features. Pearson correlations were thus used to explore the association between the cerebellar GM volumes and FC with cognitive (MoCA scores) and motor (UPDRS-III scores) functions. Correlation analyses were carried out using SPSS version 21 and statistical thresholds were set at *p* < 0.05 (two-tailed). The effect size estimate for two-group comparisons and the correlation analyses were measured by Cohen's *d*, which was implemented in GPower 3.1 (https://www.psychologie.hhu.de/).

## Results

### Demographic and Clinical Characteristics

After quality control measurements, structural imaging from 122 participants (29 EC-NC, 29 EC-CI, 30 PD-NC, and 34 PD-CI) and resting-state functional imaging from 86 participants (19 EC-NC, 21 EC-CI, 20 PD-NC, and 26 PD-CI) were analyzed. See Table 1 and Supplementary Table 2 for the demographic and clinical characteristics of these participants. The groups were matched in age, gender, educational level, and HAMD scores. Both groups of PD patients (normal cognition vs. cognitive impairment) had comparable disease duration, H&Y disease stage, daily levodopa equivalent dosage, and UPDRS scores. Finally, with regard to group stratification by cognitive status, the group of PD patients with normal cognition and that of elderly controls with normal cognition had similar MoCA scores, so did the EC-CI and PD-CI groups.

**Table 1:** Demographics and clinical characteristics of elderly controls and Parkinson's disease patients with and without cognitive impairment.

**Measures**	**EC-NC (***n*** = 29)**	**EC-CI (***n*** = 29)**	**PD-NC (***n*** = 30)**	**PD-CI (***n*** = 34)**	***P*** **-value**
Age (means ± SD)	60.59 ± 8.99	61.45 ± 8.91	57.10 ± 9.67	61.73 ± 8.71	0.172
Gender (M: F)	14:15	11:18	19:11	17:17	0.277
Education (years)	10.76 ± 2.13	9.72 ± 4.58	10.63 ± 3.76	9.62 ± 3.68	0.487
HAMD	2.72 ± 2.63	3.48 ± 2.79	4.80 ± 3.69	3.44 ± 3.69	0.067
MoCA (max, 30)	27.28 ± 1.51	21.83 ± 2.24	27.20 ± 1.77	21.56 ± 3.21	<0.001
Duration of PD (years)			3.67 ± 2.94	4.26 ± 2.86	0.663
DDE (mg/day)			331.83 ± 332.68	376.76 ± 319.45	0.578
Hoehn and Yahr stage			1.41 ± 0.46	1.56 ± 0.49	0.236
UPDRS I			6.27 ± 3.96	7.35 ± 4.77	0.330
UPDSR II			6.67 ± 3.99	7.94 ± 4.04	0.210
UPDRS III			21.53 ± 9.89	25.03 ± 12.16	0.216
UPDRS IV			0.33 ± 1.02	0.08 ± 0.51	0.224

*EC-NC, elderly controls with normal cognition; EC-CI, elderly controls with cognitive impairment; PD-NC, Parkinson disease with normal cognition; PD-CI, Parkinson disease with cognitive impairment; SD, Standard Deviation; M:F, Male: Female; HAMD, Hamilton Depression scale; MoCA, Montreal Cognitive Assessment; MMSE, Min-Mental State Examination; DDE, dopaminergic dose equivalence; UPDRS-I, Unified Parkinson's Disease Rating Scale part I: non-motor experiences of daily living; UPDRS-II, Unified Parkinson's Disease Rating Scale part II: motor experiences of daily living; UPDRS-III, Unified Parkinson's Disease Rating Scale part III: motor examination; UPDRS-IV, Unified Parkinson's Disease Rating Scale part IV: motor complications*.

### Cerebellar Volumetric Analysis

There was no significant difference in overall cerebellar lobular gray matter volume between the four different participant groups after correcting for multiple comparisons (Supplementary Figure 1). Both the CBMc and CBMm regions also showed comparable gray matter volume among these groups (Supplementary Figure 2). Lastly, the results of the voxel-based morphometry analysis of the entire cerebellum did not reveal any significant clusters between groups following FDR corrections.

### Cerebellar Resting-State FC Analysis

**Figure 1:**
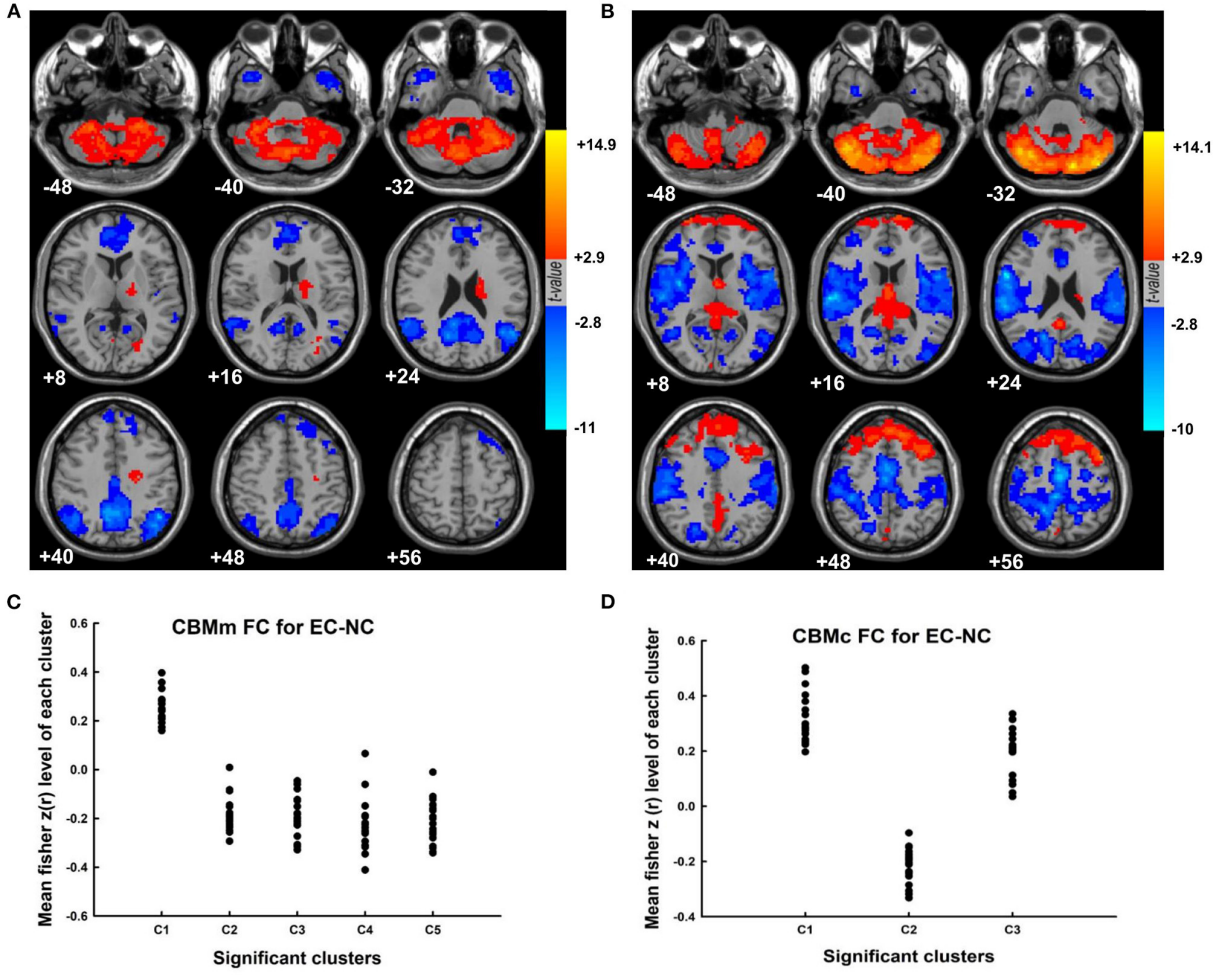
The cerebellar functional connectivity pattern in the elderly controls with normal cognition. **(A)** The group's main effect map of the “motor” cerebellar functional connectivity for elderly controls with normal cognition. **(B)** The group's main effect map of the “cognitive” cerebellar functional connectivity for elderly controls with normal cognition. **(C)** The mean fisher z(r) value within each significant cluster for the CBMm functional connectivity for individuals in elderly controls with normal cognition. The minimal cluster size was set at 556 voxels, with cluster level of *p* < 0.01. **(D)** The mean fisher z(r) value within each significant cluster for the CBMc functional connectivity for individuals in elderly controls with normal cognition. The minimal cluster size was set at 638 voxels, with cluster level of *p* < 0.01. CBMm, “motor” cerebellum, including bilateral lobules V, VI, VIIb, VIIIa, and VIIIb of the cerebellum; CBMc, “cognitive” cerebellum, including bilateral Crus I and Crus II of the cerebellum; EC-NC, elderly control with normal cognition.

The results of the “motor” and “cognitive” cerebellum to whole-brain functional connectivity in the group of elderly controls with normal cognition are shown in Figure 1 (CBMm; Figures 1A,C; CBMc; Figures 1B,D, respectively). Specifically, during resting state, healthy elderly showed positive intracerebellar connectivity and positive connectivity between CBMc and both superior frontal gyri. Meanwhile, the pattern of cerebellar FC in healthy elderly also included that the CBMm was negatively connected with the middle temporal gyrus, angular gyrus, and precuneus bilaterally, as well as the CBMc was negatively connected with the right precentral and bilateral supplementary motor area (SMA) (Supplementary Table 3). The group's main effect maps for EC-CI, PD-NC, and PD-CI as well as the specific information of the significant clusters are shown in Supplementary Figures 3–5 and Supplementary Tables 4–6, respectively.

**Figure 2:**
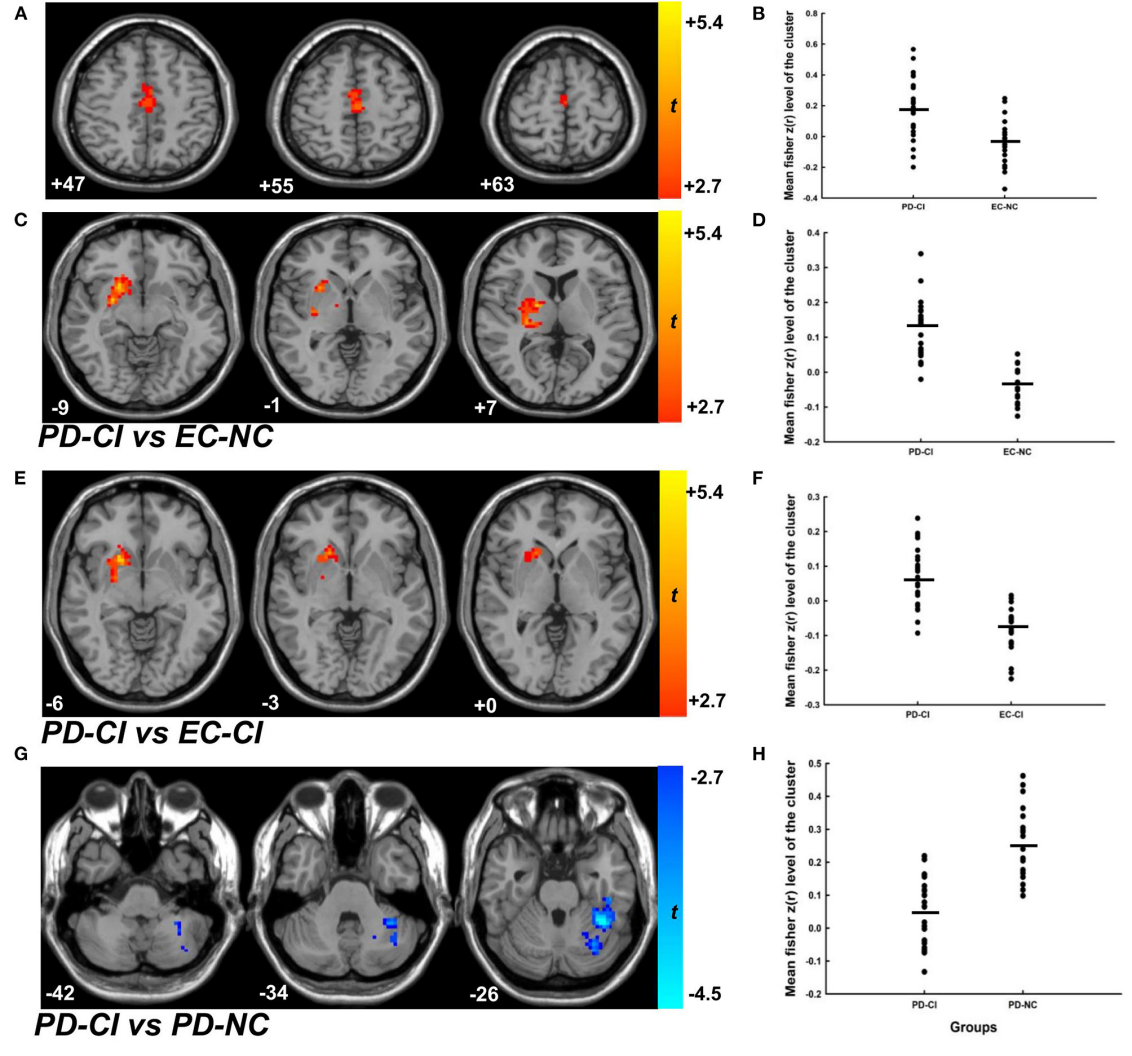
The cerebellar functional connectivity of patients with PD-CI compared to EC-NC, EC-CI as well as PD-NC groups. **(A,C)** The contrast map of CBMm functional connectivity of patients with PD-CI compared to the EC-NC group. The minimal cluster size was set at 175 voxels, with cluster level of *p* < 0.01. **(B,D)** The mean fisher z(r) value with each significant cluster for the CBMm functional connectivity between PD-CI and EC-NC groups (*p* < 0.001, Cohen *d* = 1.239; *p* < 0.001, Cohen *d* = 2.558, respectively). **(E)** The contrast map of CBMm functional connectivity of patients with PD-CI compared to the EC-CI group. The minimal cluster size was set at 177 voxels, with cluster level of *p* < 0.01. **(F)** The mean fisher z(r) value with each significant cluster for the CBMm functional connectivity between PD-CI and EC-CI groups (*p* < 0.001, Cohen *d* = 1.993). **(G)** The contrast map of CBMm functional connectivity of patients with PD-CI compared to the PD-NC group. The minimal cluster size was set at 159 voxels, with cluster level of *p* < 0.01. **(H)** The mean fisher z(r) value with each significant cluster for the CBMm functional connectivity between PD-CI and PD-NC groups (*p* < 0.001, Cohen *d* = 1.910). PD-CI, Parkinson disease with cognitive impairment; EC-NC, elderly controls with normal cognition; EC-CI, elderly controls with cognitive impairment; PD-NC, Parkinson disease with normal cognition.

ANOVA analyses on rs-fMRI data among the four groups of participants showed significant group differences in both “motor” and “cognitive” cerebellum to whole-brain functional connectivity (two significant clusters for CBMc FC: 81 voxels, *F* = 11.848, *df* = 3, *p* < 0.001; 59 voxels, *F* = 13.305, *df* = 3, *p* < 0.001; one significant cluster for CBMm FC: 101 voxels, *F* = 11.869, *df* = 3, *p* < 0.001). Further stratification analyses revealed that, compared to the healthy elderly control group, no significant difference in cerebellar FC was observed in groups with motor deficits alone (PD-NC vs. EC-NC) or with cognitive deficits alone (EC-CI vs. EC-NC). With combined motor and cognitive status, the PD-CI group revealed significant cerebellar functional connectivity compared to the EC-NC, EC-CI, and PD-NC groups (Supplementary Table 7). Compared to EC-NC group, patients with PD-CI had significantly greater CBMm connectivity with bilateral SMA (Figures 2A,B, *t* = 4.033, *df* = 43, *p* < 0.001, *d* = 1.239) as well as with the right putamen and right caudate (Figures 2C,D, *t* = 8.198, *df* = 43, *p* < 0.001, *d* = 2.558). In comparison to the EC-CI group, patients with PD-CI showed significantly greater CBMm connectivity with the right putamen and right caudate (Figures 2E,F, *t* = 6.694, *df* = 45, *p* < 0.001, *d* = 1.993). Finally, when compared with the PD-NC, patients with PD-CI showed significantly weaker connectivity between CBMc and left cerebellar VI and VIIb regions (CBMc-LC) (Figures 2G,H, *t* = −6.437, *df* = 44, *p* < 0.001, *d* =1.910), and the pattern of the results remained the same while adjusted by disease duration and the motor scores.

### Correlations Between MRI Metrics and Clinical Features

**Figure 3:**
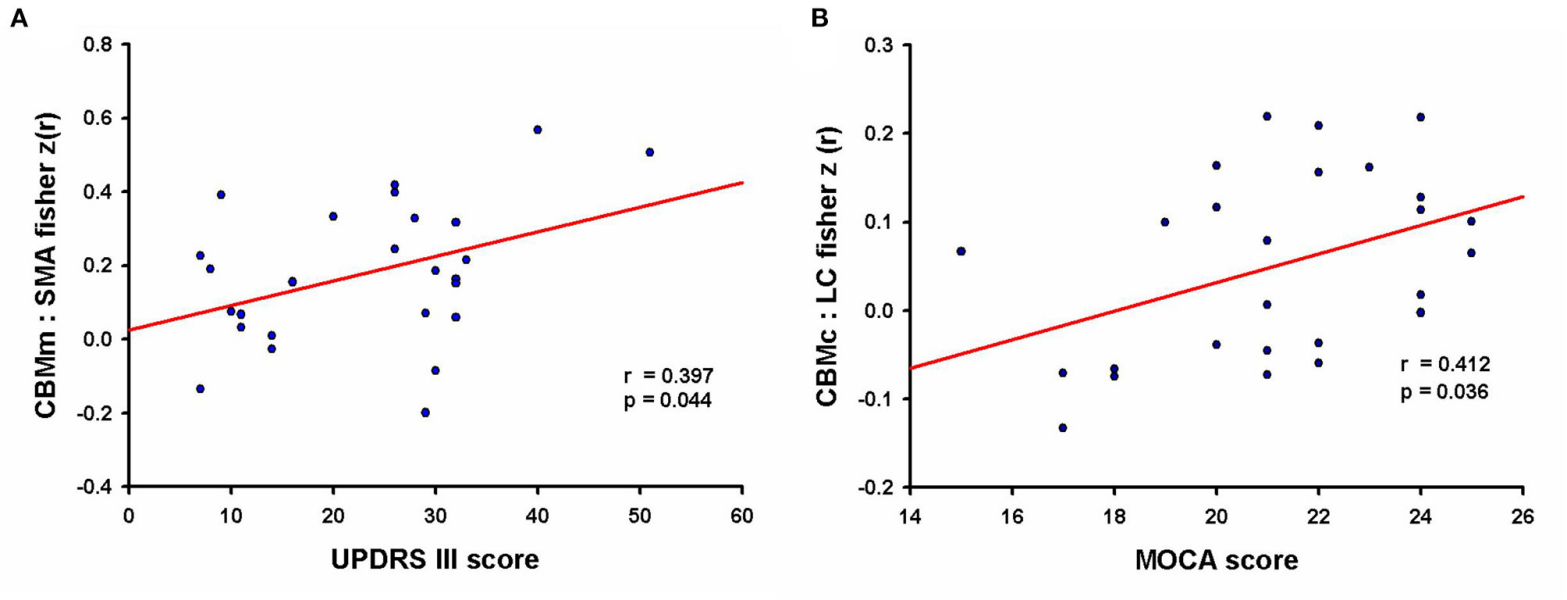
The correlation between functional connectivity and clinical features. **(A)** “Motor” cerebellum and supplementary motor area connectivity was positively correlated with the UPDRS motor scores in PD patients with cognitive impairment (effect size of Cohen *d* = 0.351); UPDRS, Unified Parkinson's Disease Rating Scale. **(B)** Negative intracerebellar connectivity was positively correlated with MoCA scores in PD patients with cognitive impairment (effect size of Cohen *d* = 0.367); MoCA, Montreal Cognitive Assessment.

The level of CBMm-SMA functional connectivity was positively correlated with the UPDRS III scores in PD patients who showed a cognitive impairment (*r* = 0.397, *p* = 0.044, *d* = 0.351, see Figure 3A). Moreover, the FC of CBMc-LC was positively correlated with the MoCA scores in PD-CI patients (*r* = 0.412, *p* = 0.036, *d* = 0.367, see Figure 3B). The cerebellar FC in EC-CI did not correlate with the MoCA scores. No other significant correlations between significant FC clusters and clinical features were observed.

## Discussion

The present study investigated relations between both structural and functional data from the cerebellum and the motor/cognitive deficits observed in patients with early PD. To do so, we stratified the group of participants into four subgroups based on their motor and cognitive status. Our results did not reveal any volumetric differences in the cerebellum among groups. Compared to the healthy elderly control subjects, however, altered cerebellar FC were observed in patients who showed both motor and cognitive deficits (i.e., early PD patients with cognitive impairment), but not those in the groups of early PD patients with normal cognition, nor elderly controls with cognitive impairment. These findings indicate that as assessed through activity at rest, the cerebellar FC may be involved in coping with both motor and cognitive demands, hence shedding additional light on our understanding of the pathophysiology of cognitive deficits in early-stage PD patients.

No significant differences among groups were observed in cerebellar lobular GM volume, nor in the results based upon the voxel-based morphometry analysis. Previous studies between patients with PD and healthy controls have shown inconsistent results on GM volumes, partly due to factors like disease duration and clinical stage (29). In line with our results, no cerebellar atrophy has previously been reported in the early-stage of the disease (30, 31). Yet, our own findings differ from those of a study by Piccinin et al. (32) who showed evidence of cerebellar atrophy in a random sample of PD patients who suffered from a tremor-dominant form of the disease. Although conjectural, such difference in pattern of results may be due to the fact, however, that all patients in the present study were scanned during “OFF” their medication phase, thus patients with prominent tremor were excluded in the data analyses due to being unable to accomplish the MRI scanning or poor data quality.

Compared to healthy controls, other researchers have reported that PD patients do reveal differences with regard to their level of cerebellar FC, ranging from no significant changes (33, 34) to significant abnormalities (20, 35–39) in connectivity during resting state. Yet such discrepancies can be attributed to the heterogeneity of PD patients regarding the clinical features or disease duration, as well as to methodological differences in MRI data analysis. Altered neural activity in the cerebellum has been previously found in PD patients with akinesia/rigidity or with freezing of gait (FOG) (35–38). However, in this study, only PD patients with H&Y ≤ 2 were included as we focused on the early stage of the disease, thus patients with FOG or serious motor deficits were not included in the current study. Moreover, recent data-driven approach studies based on rs-fMRI data showed that cerebellar FC could discriminate the patients with multiple system atrophy (MSA), but not PD, from the healthy controls, or the difference in FC between PD patients and healthy controls was not located in the cerebellum (40–42). In this study, after stratifying patients based on their cognitive status, we found that the “motor” and “cognitive” cerebellar FC changed in PD patients with cognitive impairment, but not in early PD patients with normal cognition.

Two previous studies have assessed the “motor” and “cognitive” cerebellar FC in patients with PD during resting state (20, 43). One study revealed that the cerebellar FC level changed in PD patients compared to normal control subjects, while the other showed greater cerebellar FC alterations in patients with MSA than in PD patients. However, it is worth noting that both studies included healthy participants that had a significantly better level of cognitive functioning than patients with PD. Taking the cognitive status into consideration, the present study provides novel information suggesting that the alterations in cerebellar FC in PD patients do not seem to be solely driven by motor deficits.

Additionally, a meta-analysis of task-related fMRI studies has emphasized that the cerebellum plays a significant role in the level of cognitive functioning in PD (16). Moreover, Gao et al. (44) has shown that the performance of PD patients and healthy control subjects do not differ significantly when required to execute motor and cognitive dual-task paradigms. Yet, compared to a single motor task, performance of a motor and cognitive dual task elicited enhanced cerebellar FC with motor and cognitive associated networks in PD patients, hence suggesting that the cerebellar FC resources may be engaged in integrating motor and cognitive networks when necessary. Furthermore, a correlation between the cerebellar vermal FC and cognitive deficits in PD was recently reported (45). Consistent with results from those previous studies, our group of PD patients with cognitive impairment showed altered cerebellar FC compared to PD patients with normal cognition, even when we controlled for the potential confounding effect of the disease duration and motor severity. Further correlation analysis showed that the altered cerebellar FC correlated with the MoCA score in PD patients with cognitive impairment, but not in elderly controls with cognitive impairment, thus indicating that such correlation is exclusive to PD patients with cognitive impairment. Taken together, we thus propose that cerebellar FC changes may be an underlying functional mechanism, not only for compensating for motor deficits but also for cognitive deficits in patients with early-stage of PD.

We found that the “motor” cerebellar-SMA connectivity was positively correlated with motor scores, while the negative intracerebellar connectivity (CBMc-LC) was positively correlated with the cognitive scores in PD patients with cognitive impairment. The latter findings suggest that the cerebellar FC may be implicated in the integration of motor and cognitive functions in the early-stage PD. However, the major pathological hallmarks of PD, α-synuclein deposition with Lewy bodies, are conspicuously absent in the cerebellum. Moreover, no significant atrophy in the cerebellum was observed in this study. Hence, we suggest that the altered cerebellar FC in PD-CI could be secondary to pathophysiology such as neurotransmitter dysfunction due to the dopaminergic depletion in early PD (46).

Despite the fact that we demonstrate that the cerebellar/brain FC alterations observed in PD patients are not due to motor deficits *per se*, but are also related to cognitive deficits in this group of patients, our study contains some limitations that should be considered. First, to focus on the early PD and minimize the effect of medication, we restricted our patients with H&Y ≤ 2 and scanned in the “OFF” medication phase. Thus, the results cannot be generalized to all situations. Second, one may concern about the sample size of the current study. The Cohen's *d* was provided to estimate the effect size. The results demonstrated large effect size for the group comparisons and moderate effect size for the correlation analyses. However, the weak diffuse effects should also be considered and validation studies are needed in the future (47, 48). Third, we emphasized the global cognitive deficits and did not assess the association of the specific cognitive domain with cerebellar FC in PD patients. Studies investigating the relations between a specific cognitive domain and cerebellar FC in early PD patients would help to clarify further the precise effect of cognition on the role the cerebellum plays in this disease. Finally, the current investigation is not longitudinal in nature. Future studies with a large longitudinal early-stage PD cohort would be needed to replicate the current findings and further improve our understanding on the cognitive deficits and cerebellar function in PD patients.

In conclusion, the present study demonstrates the existence of cerebellar FC changes in the resting state in early PD patients with cognitive impairment, and that these alterations in cerebellar FC correlate with both the motor UPDRS and cognitive MoCA scores. Our findings suggest that these changes in cerebellar FC may constitute an underlying compensatory mechanism for cognitive deficits in PD patients, hence indicating the interplay between motor and cognitive functioning in the early stage of PD. Yet future investigations focusing of longitudinal changes in cerebellar functional connectivity during the deterioration in cognitive functioning in PD patients are needed to provide additional insights into the interplay of motor and cognitive deficits in this neurological disorder.

## Data Availability Statement

The original contributions presented in the study are included in the article/Supplementary Material, further inquiries can be directed to the corresponding author/s.

## Ethics Statement

The studies involving human participants were reviewed and approved by the Ethic Review Committee of the Xuanwu Hospital of Capital Medical University. The patients/participants provided their written informed consent to participate in this study.

## Author Contributions

XD, TW, and PC conceived and designed the project. JS, LG, YZ, and JM collected the data of subjects. XD and YH analyzed the data. XD, JD, TW, and PC wrote and reviewed the manuscript. All authors read and approved the final manuscript.

## Conflict of Interest

The authors declare that the research was conducted in the absence of any commercial or financial relationships that could be construed as a potential conflict of interest.

## Publisher's Note

All claims expressed in this article are solely those of the authors and do not necessarily represent those of their affiliated organizations, or those of the publisher, the editors and the reviewers. Any product that may be evaluated in this article, or claim that may be made by its manufacturer, is not guaranteed or endorsed by the publisher.
