# Supplementary material for: Altered Cerebellar Resting-State Functional Connectivity in Early-Stage Parkinson's Disease Patients With Cognitive Impairment
Source: Front Neurol. 2021 Aug 25;12:678013. doi: 10.3389/fneur.2021.678013 (PMC8425347; doi:10.3389/fneur.2021.678013)
Supplement: Supplementary file 1 [file Data_Sheet_1.DOCX]

**Supplementary Methods:**

The MRI scans in patients with PD were obtained in the “off” medication state, that is after their antiparkinsonian medication had been withdrawn overnight.

The preprocessing steps for the functional MRI data were carried out using the following standard pipeline:

(1) the first 10 time points were removed; (2) the remaining 166 volumes were corrected for slice timing and realigned to the first volume for head motion correction; (3) individual functional images were co-registered to T1-weighted images; (4) the structural template was created using diffeomorphic anatomical registration through exponentiated lie algebra (DARTEL) algorithm and segmented into grey matter, white matter and cerebrospinal fluid (CSF); (5) the functional images were spatially normalized to the standard Montreal Neurological Institute (MNI) space with DARTEL segmentation; (6) Gaussian smoothing procedure was used with a 6 mm FWHM isotropic smoothing kernel; (7) linear detrend removal and temporal band-pass filtering (0.01–0.08 Hz) were implemented to remove low-frequency drift and minimize high-frequency physiological noises; (8) nuisance covariates were regressed out using multiple linear regression, including 24 head motion parameters , global mean, CSF, and white matter signals.
