# Supplementary material for: Altered Cerebellar Resting-State Functional Connectivity in Early-Stage Parkinson's Disease Patients With Cognitive Impairment
Source: Front Neurol. 2021 Aug 25;12:678013. doi: 10.3389/fneur.2021.678013 (PMC8425347; doi:10.3389/fneur.2021.678013)
Supplement: Supplementary file 2 [file Data_Sheet_2.DOCX]

**Supplementary table 1 The adopted minimal cluster sizes for multiple corrections in the functional connectivity analysis**

| **Groups** | **minimal cluster size (voxels)** | |
| --- | --- | --- |
|  | **CBMc FC** | **CBMm FC** |
| **EC-NC** | 638 | 556 |
| **EC-CI** | 694 | 627 |
| **PD-NC** | 676 | 604 |
| **PD-CI** | 710 | 642 |
| **ANOVA (4groups)** | 54 | 55 |
| **EC-CI vs EC-NC** | 140 | 169 |
| **PD-NC vs EC-NC** | 169 | 157 |
| **PD-CI vs EC-NC** | 144 | 175 |
| **PD-CI vs EC-CI** | 163 | 177 |
| **PD-CI vs PD-NC** | 159 | 139 |

CBMm, “motor” cerebellum, including bilateral lobules V, VI, VIIb, VIIIa and VIIIb of the cerebellum; CBMc, “cognitive” cerebellum, including bilateral Crus I and Crus II of the cerebellum; FC, functional connectivity; EC-NC, elderly controls with normal cognition; EC-CI, elderly controls with cognitive impairment; PD-NC, Parkinson disease with normal cognition; PD-MCI, Parkinson disease with cognitive impairment.

**Supplementary table 2 Demographics and clinical characteristics of participants in the functional** **connectivity analysis**

| **Measures** | **EC-NC (n = 19)** | **EC-CI (n = 21)** | **PD-NC (n = 20)** | **PD-CI (n = 26)** | **P-value** |
| --- | --- | --- | --- | --- | --- |
| **Age (means ± SD)** | 61.00 ± 9.31 | 59.71 ± 7.37 | 57.75 ± 10.73 | 61.11 ± 7.51 | 0.570 |
| **Gender (M: F)** | 5:14 | 9:12 | 11:9 | 11:15 | 0.345 |
| **Education(years)** | 10.89± 2.02 | 9.05 ± 5.36 | 11.65 ± 4.36 | 9.73 ± 3.65 | 0.169 |
| **HAMD** | 3.11 ± 2.73 | 3.86 ± 3.04 | 4.95 ± 3.66 | 3.96 ± 2.81 | 0.317 |
| **MoCA(max,30)** | 27.53 ± 1.65 | 22.00 ± 2.35 | 27.25 ± 1.97 | 21.77 ± 2.61 | < 0.001 |
| **Duration of PD (years)** |  |  | 3.75 ± 2.69 | 4.92 ± 1.74 | 0.080 |
| **DDE (mg/day)** |  |  | 288.75 ± 346.95 | 307.12 ± 307.17 | 0.834 |
| **Hoehn and Yahr stage** |  |  | 1.48 ± 0.47 | 1.56 ± 0.48 | 0.561 |
| **UPDRS I** |  |  | 5.90 ± 3.68 | 7.54 ± 5.27 | 0.243 |
| **UPDSR II** |  |  | 6.60 ± 3.87 | 8.89 ± 5.85 | 0.139 |
| **UPDRS III** |  |  | 22.95 ± 10.88 | 23.19 ± 11.54 | 0.943 |
| **UPDRS IV** |  |  | 0.15 ± 0.67 | 0.12 ± 0.59 | 0.853 |

EC-NC, elderly controls with normal cognition; EC-CI, elderly controls with cognitive impairment; PD-NC, Parkinson disease with normal cognition; PD-MCI, Parkinson disease with cognitive impairment; SD, Standard Deviation; M:F, Male : Female; HAMD, Hamilton Depression scale; MoCA, Montreal Cognitive Assessment; MMSE, Min-Mental State Examination; DDE, dopaminergic dose equivalence; UPDRS-I, Unified Parkinson's Disease Rating Scale part I: non-motor experiences of daily living ; UPDRS-II, Unified Parkinson's Disease Rating Scale part II: motor experiences of daily living ; UPDRS-III, Unified Parkinson's Disease Rating Scale part III: motor examination; UPDRS-IV, Unified Parkinson's Disease Rating Scale part IV: motor complications.

**Supplementary table 3 The significant clusters for the cerebellar functional connectivity in elderly controls with normal cognition**

| **ROI** | **Cluster number** | **t value** | **Cluster Size (mm^3^)** | **Brain Region** | **Peak MNI coordinates** | | |
| --- | --- | --- | --- | --- | --- | --- | --- |
|  |  |  |  |  | **X** | **Y** | **Z** |
| **CBMm** | 1 | 17.347 | 6886 | Cerebellum_6_L  Cerebelum_6_R  Cerebelum_Crus1_L | -30 | -63 | -27 |
|  | 2 | -11.866 | 3115 | Temporal_Mid_L  Frontal_Sup_Medial_L  Frontal_Sup_Medial_R | -60 | -15 | -24 |
|  | 3 | -8.937 | 1704 | Angular_R  Temporal_Mid_R  Temporal_Inf_R | 48 | -63 | 33 |
|  | 4 | -13.373 | 1656 | Precuneus_R  Precuneus_L  Cingulum_Mid_L | 12 | -54 | 33 |
|  | 5 | -11.975 | 837 | Angular_L  Parietal_Inf_L  Occipital_Mid_L | -45 | -57 | 36 |
| **CBMc** | 1 | 16.446 | 5915 | Cerebellum_Crus1_R  Cerebelum_Crus1_L  Cerebelum_6_R | 30 | -84 | -216 |
|  | 2 | -12.4845 | 14183 | Supp_Motor_Area_L  Precentral_R  Supp_Motor_Area_R | -3 | -6 | 54 |
|  | 3 | 10.156 | 2896 | Frontal_Sup_Midal_L  Frontal_Sup_R  Frontal_Sup_L | 0 | 42 | 45 |

ROI, Region of interest; CBMm, “motor” cerebellum, including bilateral lobules V, VI, VIIb, VIIIa and VIIIb of the cerebellum; CBMc, “cognitive” cerebellum, including bilateral Crus I and Crus II of the cerebellum; L, left; R, right.

**Supplementary table 4 The significant clusters for the cerebellar functional connectivity in elderly controls with cognitive impairment**

| **ROI** | **Cluster number** | **t value** | **Cluster Size (mm^3^)** | **Brain Region** | **Peak MNI coordinates** | | |
| --- | --- | --- | --- | --- | --- | --- | --- |
|  |  |  |  |  | **X** | **Y** | **Z** |
| **CBMm** | 1 | 19.028 | 7402 | Cerebellum_6_L  Cerebelum_6_R  Cerebellum_8_L  Cerebelum_8_R  Cerebelum_Crus1_L  Cerebelum_Crus1_R | 21 | -66 | -21 |
|  | 2 | -8.316 | 2550 | Temporal_Mid_R  Temporal_Sup_R  Angular_R  Frontal_Inf_Orb_R  Hippocampus_R | 33 | 24 | -15 |
|  | 3 | -10.541 | 5740 | Temporal_Mid_L  Frontal_Sup_Midial_L  Frontal_Sup_Midial_R  Angular_L  Frontal_Sup_L  Frontal_Mid_L | -48 | -66 | 36 |
|  | 4 | -7.569 | 1046 | Precuneus_L  Precuneus_R  Cinglulum_Mid_L  Cingulum_Mid_R  Cingulum_Post_L  Cingulum_Post_R | -3 | -54 | 33 |
|  | 5 | 9.0734 | 1061 | Frontal_Sup_R  Supp_Motor_Area_R  Frontal_Sup_L  Precentral_L  Precentral_R  Supp_Motor_Area_L | 24 | 6 | 51 |
|  | 6 | 6.027 | 755 | Parietal_Sup_R  Parietal_Sup_L  Precuneus_L  Postcentral_R  Precuneus_R | 15 | -60 | 72 |
| **CBMc** | 1 | 9.753 | 847 | Temporal_Mid_L  Temporal_Inf_L  Fusiform_L  Temporal_Pole_Mid_L | -60 | -33 | -9 |
|  | 2 | 22.481 | 5826 | Cerebelum_Crus1_R  Cerebelum_Crus1_L  Cerebelum_Crus2_R  Cerebelum_Crus2_L  Temporal_Inf_R  Cerebelum_6_R | -36 | -81 | -30 |
|  | 3 | 11.557 | 3499 | Frontal_Mid_L  Frontal_Sup_Midal_L  Frontal_Sup_R  Frontal_Sup_L  Frontal_Mid_R | 0 | 33 | 63 |
|  | 4 | -14.019 | 15922 | Postcentral_R  Postcentral_L  Precentral_R  Temporal_Sup_L  Temporal_Sup_R | 9 | -15 | 45 |

ROI, Region of interest; CBMm, “motor” cerebellum, including bilateral lobules V, VI, VIIb, VIIIa and VIIIb of the cerebellum; CBMc, “cognitive” cerebellum, including bilateral Crus I and Crus II of the cerebellum; L, left; R, right.

**Supplementary table 5 The significant clusters for the cerebellar functional connectivity in PD patients with normal cognition**

| **ROI** | **Cluster number** | **t value** | **Cluster Size (mm^3^)** | **Brain Region** | **Peak MNI coordinates** | | |
| --- | --- | --- | --- | --- | --- | --- | --- |
|  |  |  |  |  | **X** | **Y** | **Z** |
| **CBMm** | 1 | 17.535 | 6496 | Cerebellum_6_L  Cerebelum_6_R  Cerebelum_Crus1_L  Cerebelum_8_R  Cerebelum_Crus1_R  Cerebellum_4_5_L | -24 | -60 | -27 |
|  | 2 | -8.478 | 918 | Temporal_Mid_R  Temporal_Sup_R  Temporal_Pole_Sup_R  Temporal_Pole_Mid_R  Temporal_Inf_R  Insula_R | 63 | -15 | -15 |
|  | 3 | -9.325 | 1802 | Temporal_Mid_L  Angular_L  Temporal_Sup_L  Temporal_Inf_L  Parietal_Inf_L  Amygdala_L | -57 | -18 | -18 |
|  | 4 | -7.994 | 2804 | Frontal_Sup_Midal_L  Frontal_Sup_Midal_R  Frontal_Sup_L  Frontal_Sup_R  Frontal_Mid_L  Frontal_Med_Orb_L | 0 | 54 | 33 |
|  | 5 | -7.64 | 1246 | Precuneus_R  Precuneus_L  Calcarine_L  Cingulum_Mid_L  Cuneus_L  Calcarine_R | 6 | -60 | 27 |
|  | 6 | 6.325 | 642 | Supp_Motor_Area_R  Supp_Motor_Area_L  Frontal_Sup_R  Postcentral_L  Frontal_Sup_L  Parietal_Sup_L | 12 | -3 | 78 |
| **CBMc** | 1 | 20.781 | 6440 | Cerebelum_Crus1_L  Cerebelum_Crus1_R  Cerebelum_Crus2_R  Cerebelum_Crus2_L  Temporal_Inf_R  Cerebelum_6_R | -42 | -69 | -36 |
|  | 2 | 14.201 | 17837 | Postcentral_R  Postcentral_L  Precentral_R  Precentral_L  Frontal_Sup_R  Temporal_Sup_L | 3 | 39 | 51 |
|  | 3 | 11.432 | 938 | Precuneus_L  Cingulum_Post_L  Cingulum_Post_R  Cingulum_Mid_L  Precuneus_R  Cingulum_Mid_R | -3 | -36 | 6 |

PD, Parkinson’s disease; ROI, Region of interest; CBMm, “motor” cerebellum, including bilateral lobules V, VI, VIIb, VIIIa and VIIIb of the cerebellum; CBMc, “cognitive” cerebellum, including bilateral Crus I and Crus II of the cerebellum; L, left; R, right.

**Supplementary table 6 The significant clusters for the cerebellar functional connectivity in PD patients with cognitive impairment**

| **ROI** | **Cluster number** | **t value** | **Cluster Size (mm^3^)** | **Brain Region** | **Peak MNI coordinates** | | |
| --- | --- | --- | --- | --- | --- | --- | --- |
|  |  |  |  |  | **X** | **Y** | **Z** |
| **CBMm** | 1 | 16.785 | 10134 | Cerebellum_6_L  Cerebelum_6_R  Cerebelum_Crus1_L  Cerebelum_8_R  Cerebelum_8_L  Precuneus_L | 15 | -54 | -21 |
|  | 2 | -8.196 | 1005 | Temporal_Mid_R  Temporal_Inf_R  Temporal_Pole_Mid_R  Temporal_Sup_R  Temporal_Pole_Sup_R  Hippocampus_R | 63 | -6 | -15 |
|  | 3 | -8.588 | 5805 | Frontal_Sup_Midal_L  Temporal_Mid_L  Frontal_Sup_Midal_R  Frontal_Sup_L  Frontal_Sup_R  Frontal_Mid_L | -39 | 15 | -21 |
|  | 4 | -7.466 | 845 | Angular_L  Parietal_Inf_L  Temporal_Mid_L  Occipital_Mid_L  Parietal_Sup_L  SupraMarginal_L | -45 | -66 | 36 |
|  | 5 | -8.846 | 687 | Angular_R  Parietal_Inf_R  Temporal_Mid_R  Temporal_Sup_R  Occipital_Mid_R  SupraMarginal_R | 57 | -51 | 27 |
|  | 6 | 7.136 | 2879 | Supp_Motor_Area_R  Supp_Motor_Area_L  Precentral_R  Postcentral_L  Precentral_R  Precentral_Lobule_L | -9 | 9 | 42 |
| **CBMc** | 1 | 18.596 | 7656 | Cerebelum_Crus1_R  Cerebelum_Crus1_L  Cerebelum_Crus2_R  Cerebelum_Crus2_L  Temporal_Mid_L  Cerebelum_6_R | 9 | -84 | -24 |
|  | 2 | -11.962 | 18135 | Postcentral_R  Postcentral_L  Temporal_Mid_R  Precentral_R  Temporal_Sup_L  Temporal_Sup_R | -63 | -30 | 15 |
|  | 3 | 10.884 | 4028 | Frontal_Sup_Medial_L  Frontal_Sup_L  Frontal_Mid_L  Frontal_Sup_Medial_R  Frontal_Sup_R  Frontal_Mid_R | 6 | 27 | 60 |

PD, Parkinson’s disease; ROI, Region of interest; CBMm, “motor” cerebellum, including bilateral lobules V, VI, VIIb, VIIIa and VIIIb of the cerebellum; CBMc, “cognitive” cerebellum, including bilateral Crus I and Crus II of the cerebellum; L, left; R, right.

**Supplementary table 7 Contrasting cerebellar functional connectivity of significant clusters base on disease status and/or cognitive status**

| **ROI** | **Comparison** | **Cluster number** | **t value** | **Cluster**  **Size (mm^3^)** | **Brain Regions** | **Peak MNI coordinates** | | | **Brodmann area** |
| --- | --- | --- | --- | --- | --- | --- | --- | --- | --- |
|  |  |  |  |  |  | **X** | **Y** | **Z** |  |
|  | PD-CI vs  EC-NC | 1 | 5.41 | 315 | Putamen_R  Pallidum_R  Thalamus_R  Insula_R  Caudate_R | 30 | -18 | 24 | BA 13  BA 47 |
| **CBMm** |  | 2 | 3.93 | 184 | Supp_Motor_Area_L  Supp_Motor_Area_R  Cingulum_Mid_L  Paracentral_Lobule_L  Cingulum_Mid_R | 0 | -18 | 54 | BA 6  BA 24  BA 32  BA 31 |
|  | PD-CI vs  EC-CI | 1 | 5.41 | 184 | Putamen_R  Frontal_Inf_Orb_R  Frontal_Sup_Orb_R  Caudate_R  Frontal_Mid_Orb_R  Pallidum_R | 21 | 12 | -9 | BA 11  BA 47 |
| **CBMc** | PD-CI vs  PD-NC | 1 | -4.52 | 213 | Cerebelum_6_L  Cerebelum_Crus1_L  Fusiform_L  Temporal_Inf_L  Cerebelum_7b_L | -39 | -48 | -27 | BA 37  BA 20  BA36 |

ROI, Region of interest; CBMm, “motor” cerebellum, including bilateral lobules V, VI, VIIb, VIIIa and VIIIb of the cerebellum; CBMc, “cognitive” cerebellum, including bilateral Crus I and Crus II of the cerebellum; L, left; R, right; PD-CI, Parkinson disease with cognitive impairment; VS: versus; EC-NC, elderly controls with normal cognition; EC-CI, elderly controls with cognitive impairment; PD-NC, Parkinson disease with normal cognition.
