# Supplementary material for: Altered Cerebellar Resting-State Functional Connectivity in Early-Stage Parkinson's Disease Patients With Cognitive Impairment
Source: Front Neurol. 2021 Aug 25;12:678013. doi: 10.3389/fneur.2021.678013 (PMC8425347; doi:10.3389/fneur.2021.678013)
Supplement: Supplementary file 3 [file Data_Sheet_3.docx]

**Supplementary Figure 1** The cerebellar lobular gray matter volume among four groups of participants.


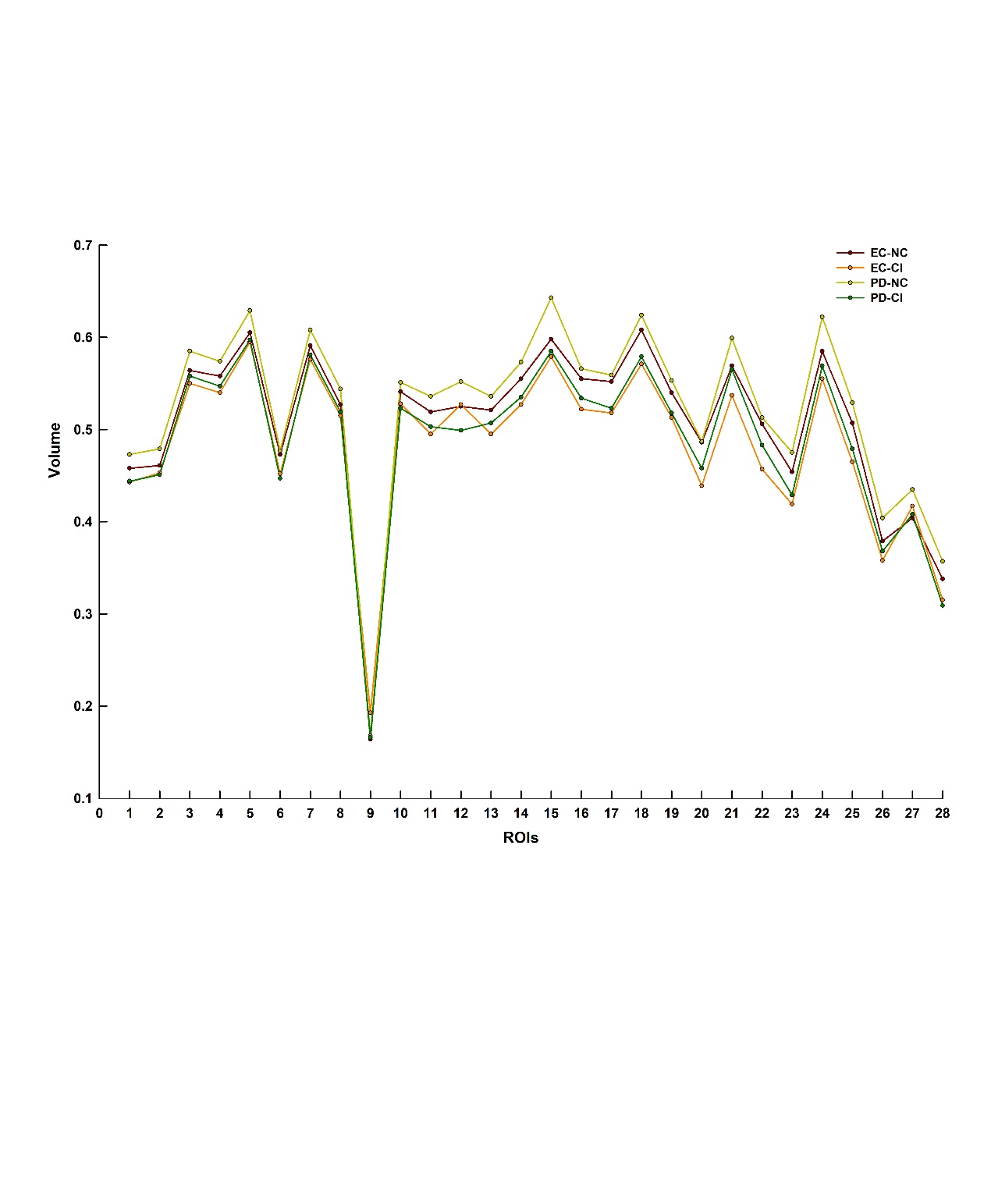


EC-NC, elderly controls with normal cognition; EC-CI, elderly controls with cognitive impairment; PD-NC, Parkinson disease with normal cognition; PD-CI, Parkinson disease with cognitive impairment

**Supplementary Figure 2** The gray matter volume of the “CBMc” and “CBMm” among the four groups of participants.


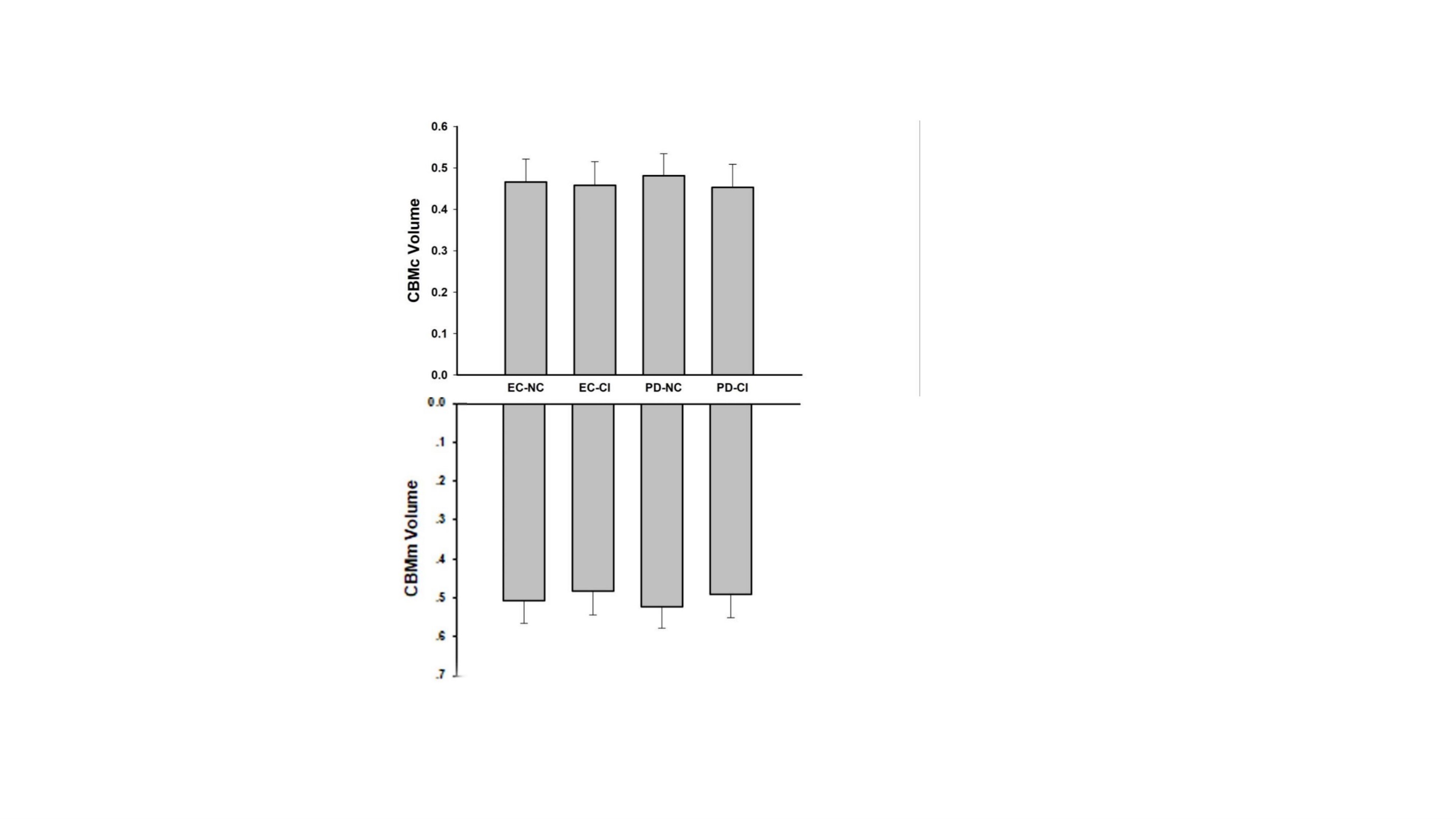


EC-NC, elderly controls with normal cognition; EC-CI, elderly controls with cognitive impairment; PD-NC, Parkinson disease with normal cognition; PD-CI, Parkinson disease with cognitive impairment; CBMm, “motor” cerebellum, including bilateral lobules V, VI, VIIb, VIIIa and VIIIb of the cerebellum; CBMc, “cognitive” cerebellum, including bilateral Crus I and Crus II of the cerebellum. There was no significance of gray matter volume for the CBMc and CBMm among the four groups of participants.

**Supplementary Figure 3** The cerebellar functional connectivity pattern in the elderly controls with cognitive impairment.


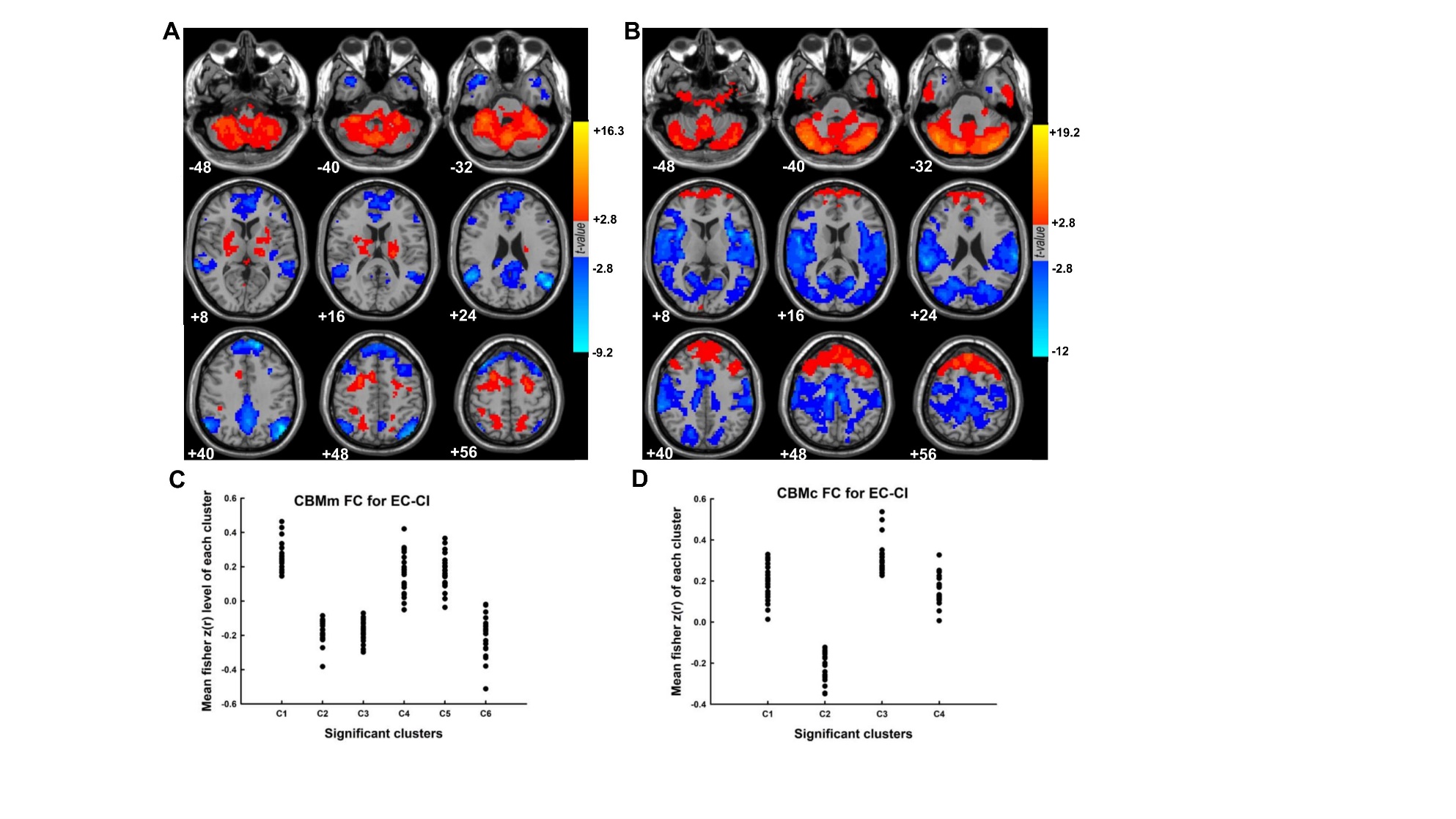


**(A)** The group’s main effect map of “motor” cerebellar functional connectivity for elderly controls with cognitive impairment. **(B)** The group’s main effect map of “cognitive” cerebellar functional connectivity for elderly controls with cognitive impairment. **(C)** The mean fisher z(r) value within each significant cluster for the CBMm functional connectivity for individuals in elderly controls with cognitive impairment. The minimal cluster size was set at 627 voxels, with cluster level of P < 0.01. **(D)** The mean fisher z(r) value within each significant cluster for the CBMc functional connectivity for individuals in elderly controls with cognitive impairment. The minimal cluster size was set at 694 voxels, with cluster level of P < 0.01. CBMm, “motor” cerebellum, including bilateral lobules V, VI, VIIb, VIIIa and VIIIb of the cerebellum; CBMc, “cognitive” cerebellum, including bilateral Crus I and Crus II of the cerebellum; EC-CI, elderly control with cognitive impairment.

**Supplementary Figure 4** The cerebellar functional connectivity pattern in PD patients with normal cognition


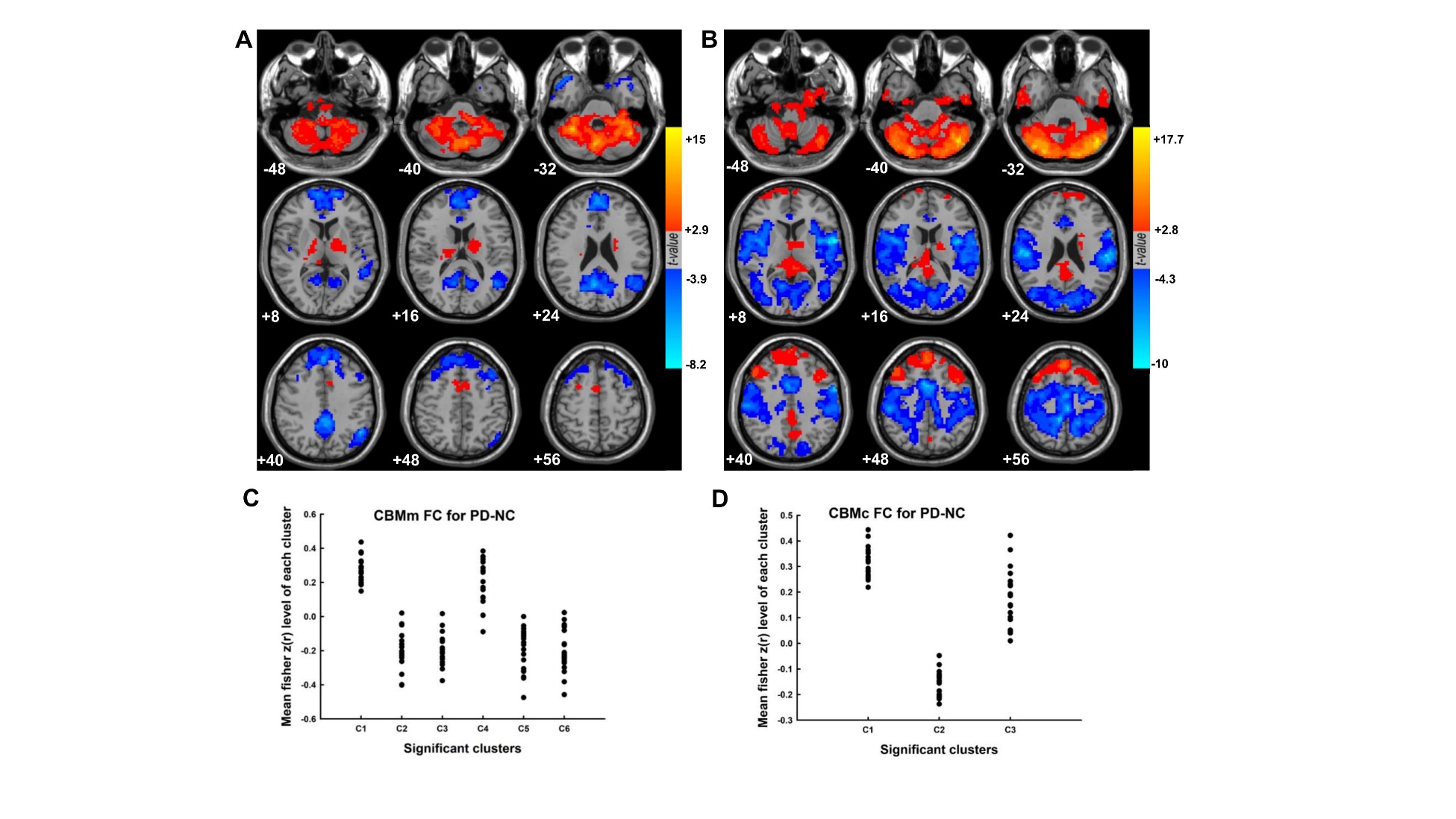


**(A)** The group’s main effect map of “motor” cerebellar functional connectivity for PD patients with normal cognition. **(B)** The group’s main effect map of “cognitive” cerebellar functional connectivity for PD patients with normal cognition. **(C)** The mean fisher z(r) value within each significant cluster for the CBMm functional connectivity for individuals in PD patients with normal cognition. The minimal cluster size was set at 604 voxels, with cluster level of P < 0.01. **(D)** The mean fisher z(r) value within each significant cluster for the CBMc functional connectivity for individuals in PD patients with normal cognition. The minimal cluster size was set at 676 voxels, with cluster level of P < 0.01. CBMm, “motor” cerebellum, including bilateral lobules V, VI, VIIb, VIIIa and VIIIb of the cerebellum; CBMc, “cognitive” cerebellum, including bilateral Crus I and Crus II of the cerebellum; PD, Parkinson’s disease; PD-NC, PD patients with normal cognition.

**Supplementary Figure 5** The cerebellar functional connectivity pattern in PD patients with cognitive impairment


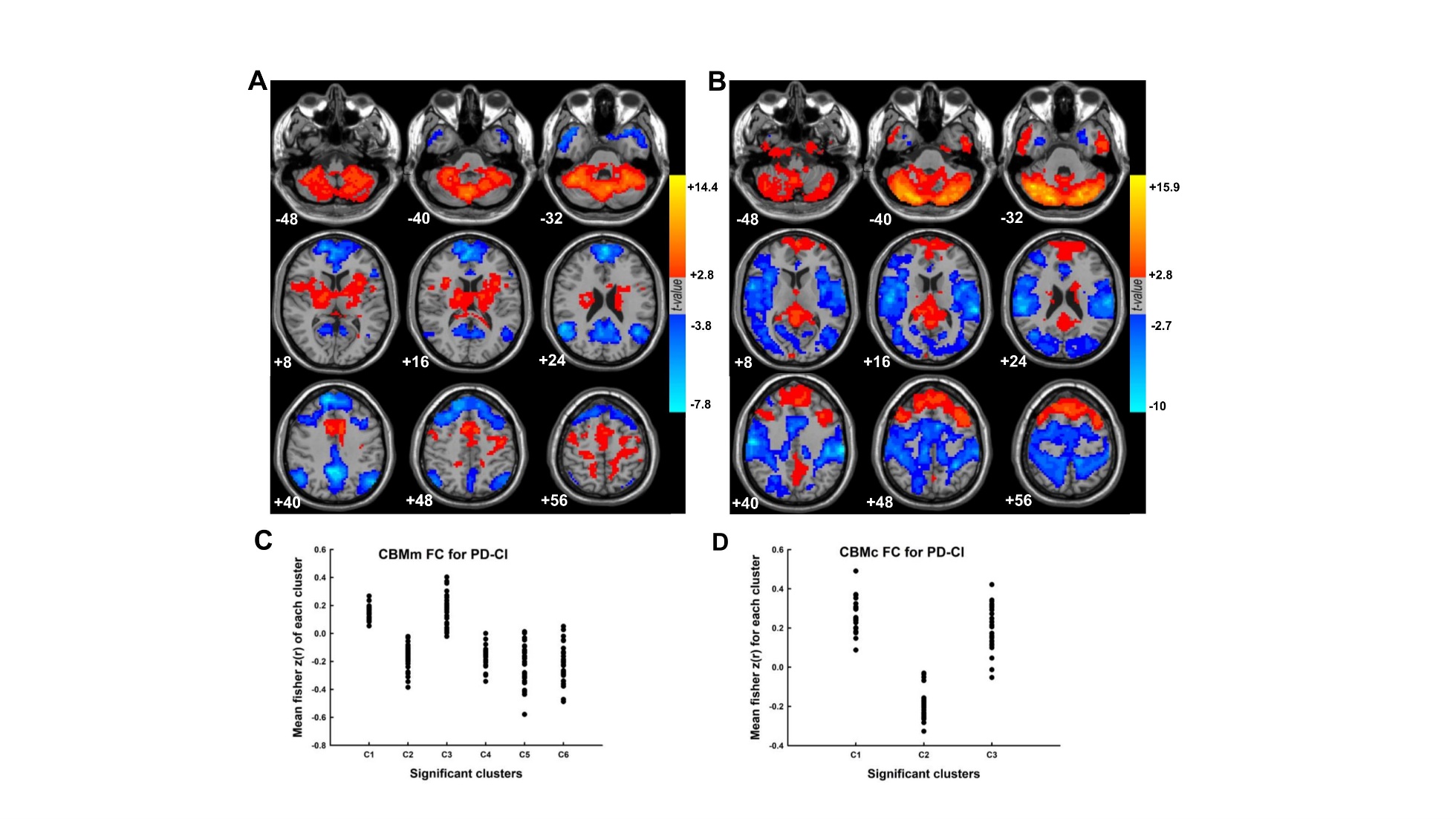


**(A)** The group’s main effect map of “motor” cerebellar functional connectivity for PD patients with cognitive impairment. **(B)** The group’s main effect map of “cognitive” cerebellar functional connectivity for PD patients with cognitive impairment. **(C)** The mean fisher z(r) value within each significant cluster for the CBMm functional connectivity for individuals in PD patients with cognitive impairment. The minimal cluster size was set at 642voxels, with cluster level of P < 0.01. **(D)** The mean fisher z(r) value within each significant cluster for the CBMc functional connectivity for individuals in PD patients with cognitive impairment. The minimal cluster size was set at 710 voxels, with cluster level of P < 0.01. CBMm, “motor” cerebellum, including bilateral lobules V, VI, VIIb, VIIIa and VIIIb of the cerebellum; CBMc, “cognitive” cerebellum, including bilateral Crus I and Crus II of the cerebellum; PD, Parkinson’s disease; PD-CI, PD patients with cognitive impairment.
